# Fibroblast Growth Factor 21 Deficiency Attenuates Experimental Colitis-Induced Adipose Tissue Lipolysis

**DOI:** 10.1155/2017/3089378

**Published:** 2017-05-11

**Authors:** Liming Liu, Cuiqing Zhao, Ying Yang, Xiaoxia Kong, Tuo Shao, Li Ren, Xinyu Zhuang, Baishuang Yin, Gerald Dryden, Craig McClain, Weimin Luan, Wenke Feng

**Affiliations:** ^1^College of Animal Science and Technology, Key Laboratory of Animal Production and Production Quality and Security, Ministry of Education, Jilin Agricultural University, Changchun, Jilin 130118, China; ^2^Departments of Medicine, Pharmacology and Toxicology, University of Louisville School of Medicine, Louisville, KY 40202, USA; ^3^College of Animal Science and Technology, Key Lab of Preventive Veterinary Medicine in Jilin Province, Jilin Agricultural Science and Technology University, Jilin, Jilin 132101, China; ^4^Institute of Virology, Wenzhou University, Wenzhou, Zhejiang 325027, China; ^5^Schools of Pharmacy and Basic Medical Sciences, Wenzhou Medical University, Wenzhou, Zhejiang 325027, China; ^6^First Affiliated Hospital of Xi'an Jiaotong University, Xi'an, Shaanxi 710061, China; ^7^Institute of Military Veterinary Institute, Academy of Military Medical Science, Changchun, Jilin 130122, China; ^8^Alcohol Research Center, University of Louisville School of Medicine, Louisville, KY 40202, USA; ^9^Robley Rex VA Medical Center, Louisville, KY 40206, USA

## Abstract

**Aims:**

Nutrient deficiencies are common in patients with inflammatory bowel disease (IBD). Adipose tissue plays a critical role in regulating energy balance. Fibroblast growth factor 21 (FGF21) is an important endocrine metabolic regulator with emerging beneficial roles in lipid homeostasis. We investigated the impact of FGF21 in experimental colitis-induced epididymal white adipose tissue (eWAT) lipolysis.

**Methods:**

Mice were given 2.5% dextran sulfate sodium (DSS) ad libitum for 7 days to induce colitis. The role of FGF21 was investigated using antibody neutralization or knockout (KO) mice. Lipolysis index and adipose lipolytic enzymes were determined. In addition, 3T3-L1 cells were pretreated with IL-6, followed by recombinant human FGF21 (rhFGF21) treatment; lipolysis was assessed.

**Results:**

DSS markedly decreased eWAT/body weight ratio and increased serum concentrations of free fatty acid (FFA) and glycerol, indicating increased adipose tissue lipolysis. eWAT intracellular lipolytic enzyme expression/activation was significantly increased. These alterations were significantly attenuated in FGF21 KO mice and by circulating FGF21 neutralization. Moreover, DSS treatment markedly increased serum IL-6 and FGF21 levels. IL-6 pretreatment was necessary for the stimulatory effect of FGF21 on adipose lipolysis in 3T3-L1 cells.

**Conclusions:**

Our results demonstrate that experimental colitis induces eWAT lipolysis via an IL-6/FGF21-mediated signaling pathway.

## 1. Introduction

Inflammatory bowel disease (IBD) is a lifestyle disease that encompasses many inflammatory disorders, particularly ulcerative colitis (UC) and Crohn's disease (CD) [1–3]. Weight loss is detected frequently among patients with IBD, and its pathophysiology is complex and multifactorial. In the active phase, there is a decrease in the oral intake of nutrients because of abdominal pain and anorexia. The mucosal inflammation and associated diarrhea lead to a loss of absorption of nutrients such as protein, minerals, electrolytes, and trace elements. Alterations in energy metabolism may result in increased resting energy expenditure and lipid oxidation in IBD patients [4, 5]. Previous studies shown that patients had fat mass depletion especially in the active phase occurred both in CD and UC patients [6]. Moreover, patients with inactive CD have lower fat mass and higher lipid oxidation rates than healthy controls [7].

In mammals, white adipose tissue (WAT) is central to lipid regulation and a major source of metabolites, facilitating both the storage of fatty acids as neutral lipids within the lipid droplets (LDs) of adipocytes and regulating the release of fatty acids in response to both acute and chronic stimuli. The intracellular degradation of triglyceride (TG) is catalyzed by a cascade of lipolytic enzymes. Perilipin (PLIN) is a lipid droplet-associated protein that controls TG storage and hydrolysis in adipocytes [8]. Three enzymes have been identified involving in the complete hydrolysis of TG molecules in cellular lipid stores. The committed enzyme catalyzing the first and rate-limiting step of TG hydrolysis is adipose triglyceride lipase (ATGL), which hydrolyzes TG to generate diglyceride (DG) and free fatty acids [9, 10]. The next step is catalyzed by hormone-sensitive lipase (HSL), a multifunctional enzyme that hydrolyzes various acylesters, including TG, DG, and monoglyceride (MG) [11]. The final step involves monoglyceride lipase (MGL), which degrades MG into glycerol and free fatty acids [12]. In addition, protein kinase A (PKA) activates HSL and PLIN by phosphorylation [13, 14], and phosphorylation of PLIN is required for activated HSL translocation from the cytosol to the surface of the lipid droplet [15].

While it is recognizable that adipose tissue lipolysis-mediated energy metabolism plays an important role in IBD, the underlying mechanism is not fully understood. Fibroblast growth factor 21 (FGF21), a member of the FGF superfamily, has emerged as an important metabolic regulator. FGF21 is a hormone mainly expressed in the liver, where it is induced by states of nutrient stress. FGF21 transgenic mice showed a promotion of adipose tissue lipolysis and upregulated lipase expression in WAT [16]. We previously reported that FGF21 mediates alcohol-induced adipose tissue lipolysis [17].

We hypothesized that FGF21 plays an important role in adipose tissue lipolysis in animals during acute colitis to regulate the local supply of energy. In the present study, we analyzed the effect of FGF21 on adipose tissue lipolysis in a murine model of DSS-induced colitis and in 3T3-L1 adipocytes. We showed that absence of FGF21 attenuates experimental colitis-induced adipose tissue lipolysis, and the effect may be mediated by IL-6 pathway.

## 2. Materials and Methods

### 2.1. Animals and Experimental Groups

Ten- to 12-week-old FGF21 knockout (KO) and C57BL/6 (WT) male mice were housed in a conventional animal room. All animal procedures were approved by the Institutional Animal Care and Use Committee (IACUC) of University of Louisville prior to the start of the study. FGF21-KO mice were provided by Dr. Steve Kliewer [18]. The mice were subjected to a 12 : 12 hour light-dark cycle in low-stress conditions with free access to food and water ad libitum. FGF21-KO mice were assigned to (1) untreated and (2) DSS groups, while WT mice were assigned to (1) untreated, (2) DSS, and (3) DSS + anti-FGF21 antibody administration groups. There was no significant difference between the body weights of FGF21-KO and WT mice as measured at the beginning of the study.

### 2.2. Induction of Experimental Colitis and FGF21 Antibody Administration

Experimental colitis was induced in FGF21-KO and WT mice. These mice received 2.5% dextran sodium sulfate (DSS; MW 36,000–50,000, MP Biomedicals, Solon, OH, USA) in drinking water for 7 days. In some mice, monoclonal rabbit-anti-mouse FGF21 antibody (abFGF21, R&D Systems Inc., Minneapolis, USA) was administered at a dose of 5 mg/kg bodyweight via intraperitoneal injection every other day starting from day 3 after colitis was induced. Control mice received same dose of rabbit IgG (nsIgG, Santa Cruz Biotechnology, Dallas, TX, USA).

### 2.3. Blood Collection and Metabolite Measurements

Human studies. Written informed consent was obtained from all participants with IBD. Healthy controls were age-, sex-, and BMI-matched to subjects with IBD. All experiments were conducted in accordance with the guidelines of human research and were approved by Clinical Research Ethics Committees of the University of Louisville, KY, USA.

All mice were euthanized on day 7. Blood and epididymal white adipose tissue (eWAT) samples were harvested from the experimental animals following euthanasia. Human and mice blood were obtained and centrifuged at 1500 rpm for 30 minutes at 4°C. The serum was collected and stored at −80°C until any experimentation. Serum free fatty acid (FFA; Wako Chemicals, Richmond, VA, USA) and glycerol (Cayman, Ann Arbor, Michigan, USA) levels were measured using commercial kits closely following the manufacturer's instructions. FGF21 concentrations in the serum were measured using an ELISA kit (R&D Systems Inc., Minneapolis, MN, USA).

### 2.4. Protein Expression and Phosphorylation Analysis

eWAT was homogenized in RIPA buffer supplemented with protease and phosphatase inhibitors. Homogenates were then centrifuged at 14,000 rpm for 15 minutes, and supernatants were collected for protein analysis. Protein concentration in the supernatant was determined using BCA (bicinchoninic acid) protein assay. Western blot was performed to detect p-HSL, HSL, ATGL, PLIN, (Cell Signaling Technologies), and *β*-actin (Santa Cruz Biotechnology) according to the protocol described previously [17]. Blots were scanned using a Bio-Rad Imaging System (Image Lab™ Upgrade for ChemiDoc™ XRS+ System #170-8299). All specific bands were quantified with the Automated Digitizing System (Image Lab 4.1 programs). Results are representative of three independent experiments.

### 2.5. Cell Culture and Treatment

3T3-L1 cells were cultured and maintained and differentiated using a previously described method [17]. Briefly, the cells were grown in DMEM/high-glucose medium (Gibco, Grand Island, NY, USA) supplemented with 10% newborn calf serum (NCS; Gibco) in a 5% CO_2_ environment. Medium was changed every 2 days. Differentiation was induced 2 days postconfluency by the addition of DMEM containing 10% fetal bovine serum (FBS; Gibco) and dexamethasone (1 *μ*M; Sigma, St. Louis, MO, USA), 3-isobutyl-1-methylxantine (IBMX) (0.5 mM; Sigma), and insulin (1 mg/l; Sigma) for 2 days. The cells were further incubated with DMEM/FBS supplemented with insulin for an additional 2 days. Fully differentiated adipocytes were maintained in DMEM/FBS at 37°C in 5% CO_2_ and used 9–14 days after the initiation of differentiation. When more than 90% of the cells showed morphological and biochemical properties of adipocytes, the cells were used for experiments. After overnight incubation in serum-free DMEM, the 3T3-L1 adipocytes were pretreated with 20 ng/mL recombinant mouse IL-6 protein (R&D System Inc., Minneapolis, MN, USA) for 18 h, followed by a treatment with 1 mg/ml recombinant human FGF21 (rhFGF21) for 2 h.

### 2.6. Statistical Analysis

Two-way ANOVA with Bonferroni posttest, or One-way ANOVA with Tukey posttest, or two-tailed unpaired Student's *t*-test were used for the determination of statistical significance of the data where they were appropriate. All statistical analyses were performed with GraphPad Prism Software Version 5 (GraphPad Software Inc., San Diego, CA, USA). For animal studies, each experimental group had seven mice. For cell culture studies, experiments were repeated three times in triplicate for each experiment. Results are expressed as mean ± SEM. Differences between groups were considered significant at ^∗^*p* < 0.05, ^∗∗^*p* < 0.01, and ^∗∗∗^*p* < 0.001.

## 3. Results

### 3.1. FGF21 Level Was Elevated during Experimental Colitis

**Figure 1:**
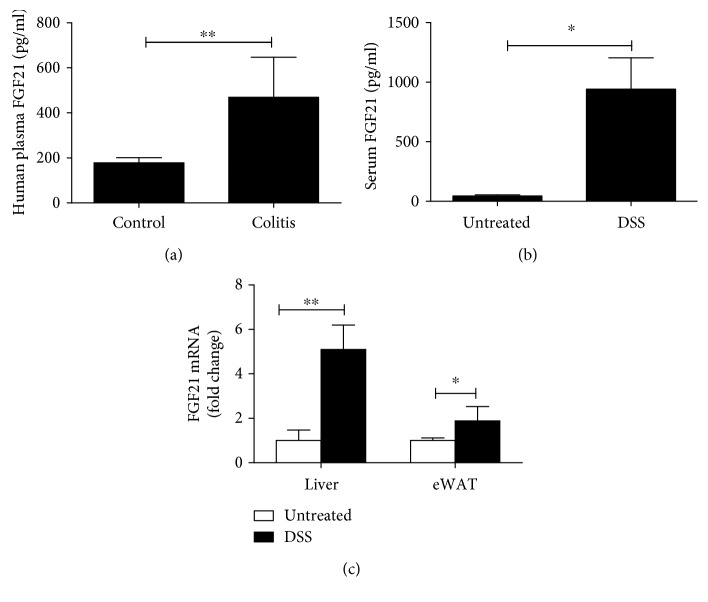
IBD in patients and experimental colitis increase FGF21 expression. (a) Serum levels of FGF21 in inflammatory bowel disease (IBD) patients. C57BL/6J mice were treated with 2.5% dextran sodium sulfate (DSS) for 7 days as described in the Materials and Methods. (b) Serum FGF21 protein levels. (c) mRNA levels of FGF21 in the liver and eWAT in WT mice.

Previous studies have shown that serum FGF21 levels are increased by inflammatory stimuli [19, 20]. Serum FGF21 levels in IBD patients were significantly higher compared to healthy controls (Figure 1(a)). We examined whether increased FGF21 in IBD patients could be reproduced in murine experimental models of colitis. As shown in Figure 1(b), serum FGF21 levels increased 20-fold approximately in mice with experimental colitis compared to normal control mice. To determine the source of FGF21, we examined FGF21 expression in two major metabolic tissues including liver and adipose tissues. A markedly increase in FGF21 gene expression in both the liver and eWAT was observed, as shown in Figure 1(c). These data demonstrate that experimental colitis increases systemic FGF21 level in mice, which agrees with our preliminary results in IBD patients. These data suggest a possible role of FGF21 in IBD.

### 3.2. FGF21 Deficiency Markedly Reduces Body Weight Loss and eWAT Lipolysis

**Figure 2:**
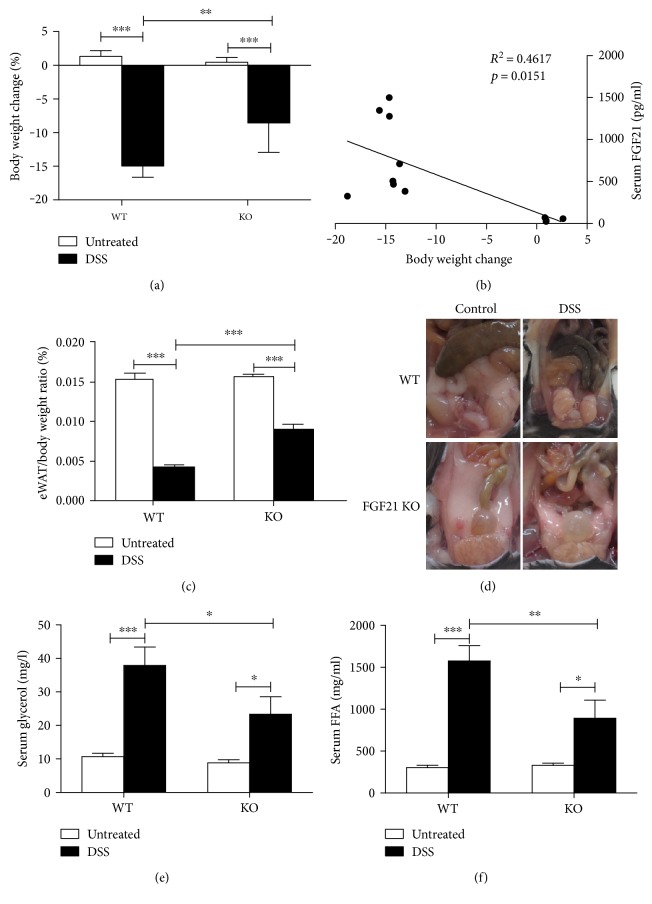
FGF21 deficiency markedly reduces experimental colitis-induced body weight loss and eWAT lipolysis. FGF21-KO mice and C57BL/6J mice were treated with 2.5% dextran sodium sulfate (DSS) for 7 days as described in the Materials and Methods. (a) Body weight change. (b) Linear correlation of the body weight change and serum FGF21 levels. (c) eWAT/body weight ratio. (d) Adipocyte size image. (e) Serum glycerol. (f) Serum FFA.

To determine whether FGF21 plays a role in body weight loss and eWAT lipolysis, global FGF21-KO mice were subjected to DSS as described in the Materials and Methods. The body weight was decreased approximately 15% in WT mice by DSS treatment, but only an 8.5% reduction was observed in FGF21-KO mice (Figure 2(a)). Interestingly, the serum FGF21 concentrations were positively correlated with the body weight change (Figure 2(b)). In addition, experimental colitis markedly reduced eWAT/body weight ratio and eWAT adipocyte size in WT mice, but not in FGF21-KO mice (Figures 2(c) and 2(d)). These results suggest that FGF21-KO mice are resistant to colitis-induced body weight loss and eWAT lipolysis. Next, we measured glycerol and FFA levels in the circulation as indices of lipolysis. As expected, serum glycerol (Figure 2(e)) and FFA (Figure 2(f)) levels were markedly increased in WT mice. However, these elevations were attenuated in FGF21-KO mice.

### 3.3. FGF21 Ablation Attenuates Lipolysis by Downregulation of ATGL Expression and HSL Phosphorylation

**Figure 3:**
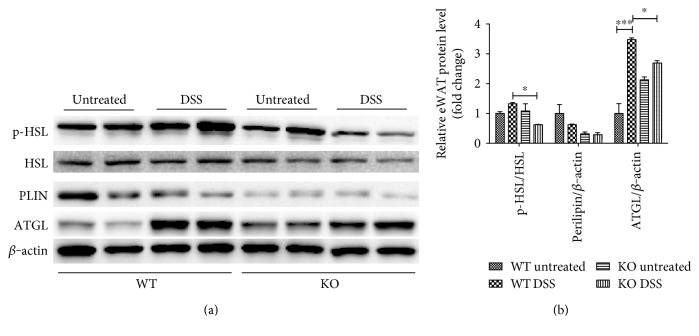
FGF21-KO mice have reduced expression and activity of lipolytic enzyme. Mice were fed as described in the Materials and Methods. (a) Proteins in eWAT were analyzed by Western blotting. (b) The quantification of protein bands in (a) by densitometry analysis.

We next examined the protein levels and the activation of the enzymes involved in regulation of lipolysis in eWAT. As shown in Figure 3, there was no significant difference in PLIN and HSL expression between untreated and DSS-treated mice. DSS treatment slightly increased the phosphorylation levels of HSL at Ser660 in WT mice, which was significantly reduced in FGF21-KO mice. In addition, DSS treatment caused a marked increase in ATGL protein level in WT mice which was attenuated in FGF21-KO mice.

### 3.4. FGF21 Neutralization Reduces Experimental Colitis-Induced eWAT Lipolysis

**Figure 4:**
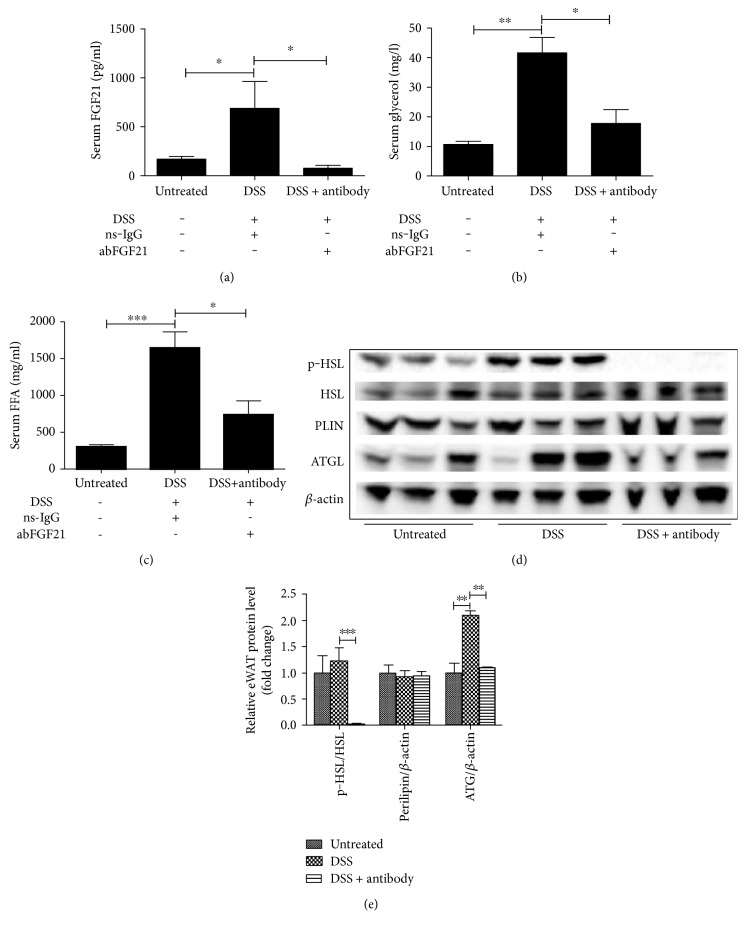
FGF21 antibody administration inhibited experimental colitis-induced eWAT lipolysis. C57BL/6J mice were treated with 2.5% dextran sodium sulfate (DSS) for 7 days, as described in the Materials and Methods. The mice in the WT DSS group were injected with FGF21 antibody (abFGF21) at 5 mg/kg body weight every other day starting from day 3 after colitis was induced. (a) Serum FGF21 protein levels. (b) Serum glycerol. (c) Serum FFA. (d) Immunoblot analysis of eWAT phosphor-HSL, HSL, PLIN, and ATGL protein levels. (e) The quantification of the immunoblot bands.

The role of FGF21 was further determined using antibody neutralization. The monoclonal rabbit-anti-mouse FGF21 antibody was injected as described in Materials and Methods. As shown in Figure 4(a), nonspecific IgG did not affect serum FGF21 level compared to the values of in Figure 1(a), while it was neutralized by the anti-FGF21 antibody. Interestingly, experimental colitis-induced glycerol (Figure 4(b)) and FFA (Figure 4(c)) elevations were inhibited by the antibody. Next, we examined whether neutralization of circulating FGF21 could also inhibit colitis-induced lipolytic enzyme activation in eWAT. As expected, DSS-induced HSL phosphorylation and ATGL expression in eWAT were significantly decreased by FGF21 neutralization. In addition, FGF21 antibody injection did not affect PLIN expression (Figures 4(d) and 4(e)). These results further demonstrate that colitis-induced adipose tissue lipolysis is mediated by FGF21 signaling in mice.

### 3.5. IL-6 Mediates the Prolipolysis Function of FGF21 in 3T3-L1 Adipocytes

**Figure 5:**
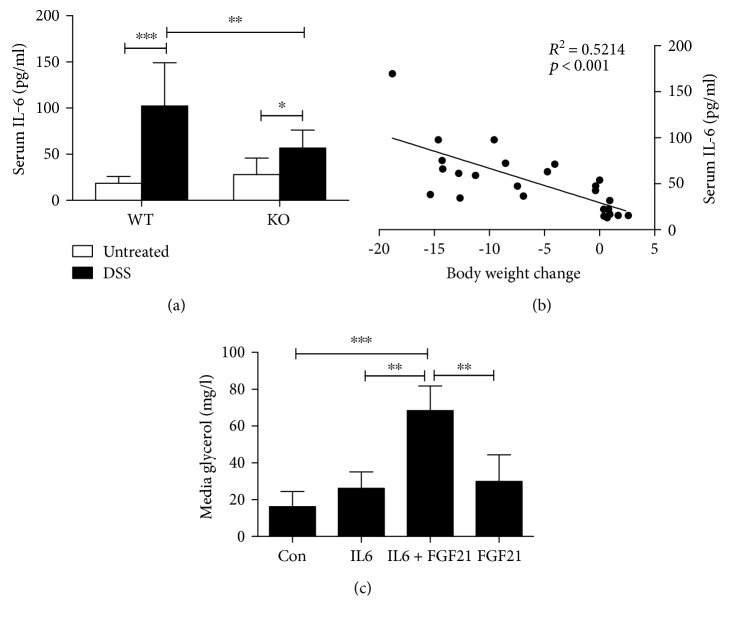
IL-6 mediates the prolipolysis function of FGF21. Mice were fed as described in the Materials and Methods. (a) Serum IL-6 levels. (b) Linear correlation of the body weight change and serum IL-6 levels. (c) 3T3-L1 adipocytes were pretreated with or without IL-6 for 18 h, followed by treatment with rhFGF21 for 2 h. Median glycerol were determined as described in Materials and Methods.

Higher serum levels of IL-6 have been reported in colitis patients and DSS-induced experimental colitis [21, 22], and it positively correlated with disease severity in colitis [23]. As shown in Figure 5(a), serum IL-6 levels were markedly increased in WT mice compared to the KO mice in response to DSS treatment. Furthermore, the serum IL-6 levels were positively correlated with the body weight change (Figure 5(b)). We found that, as early as DSS treatment at day 3, serum IL-6 was elevated and significantly increased at day 7. However, serum FGF21 elevations started at day 5, which is later than IL-6 induction (data not shown). We postulated that IL-6 is involved in the FGF21-mediated adipose tissue lipolysis. To explore this possibility, we examined the median glycerol concentration in fully differentiated 3T3-L1 cells exposed to IL-6 and/or rhFGF21. As shown in Figure 5(c), basal glycerol in either IL-6- or FGF21-treated adipocytes was not changed compared to that in untreated control cells. However, rhFGF21 significantly increased median glycerol concentrations in 3T3-L1 cells pretreated with IL-6 for 18 hours. These data suggest that IL-6 is required for FGF21-mediated adipose tissue lipolysis.

## 4. Discussion

IBD is associated with nutritional deficiencies, and alterations in energy metabolism may result in increased lipid utilization in IBD patients. Adipose tissue lipolysis is a critical pathway for the maintenance of energy homeostasis through degradation of triglyceride and release of fatty acid into the circulation. The adipose tissue depots can be altered due to inflammatory such as CD [24]. As previously described, both CD and UC patients had fat mass depletion [6]. In addition, DSS-induced experimental colitis presented lower epididymal white adipose tissue to body weight ratio and smaller adipocyte size [25]. The precise mechanisms regulating WAT lipolysis in colitis are not fully understood.

FGF21 is a metabolic regulator. Previous studies have shown that FGF21 plays a role in adipose tissue lipolysis under metabolic stress conditions. However, controversial results have been documented that some studies suggested that FGF21 stimulates lipolysis [16], while other studies showed that FGF21 was likely a negative factor in adipose lipolysis [26]. The adipose tissue lipolysis under inflammatory condition has not yet been elucidated. Our results showed that FGF21 induction is a negative factor in adipose lipolysis in DSS-induced colitis, which is known as an inflammatory-associated disorder.

Adipose tissue lipolysis is an exquisitely controlled process [27, 28]. Adipose PKA activation mediates the activation of HSL by phosphorylation at Ser 660. PLIN is a coating protein that binds to the surface of lipid droplets and appears to be essential for lipid degradation mediated by HSL [29]. Another major lipolytic enzyme, ATGL, favors TG substrates and is a rate-determining enzyme for lipolysis in adipose tissue [9, 30]. Our results supported this notion as the levels of p-HSL, ATGL, and PLIN were increased by DSS-induced colitis in eWAT.

Previous studies showed that inflammatory stimuli increased FGF21 levels [19], which is confirmed in the current study in an experimental IBD model. Circulating FGF21 was significantly elevated by DSS at days 5 and 7. This elevation implies a possible role of FGF21 in DSS-induced colitis. Using FGF21 knockout mice, our study demonstrated that experimental colitis-associated eWAT loss is attenuated when FGF21 is absent. This notion was further demonstrated by administration of an anti-FGF21 antibody to neutralize the circulating FGF21. The attenuation of DSS-induced lipolysis in FGF21 deactivation was associated with the decrease in p-HSL, ATGL, and PLIN expression in adipose tissue.

How does FGF21 contribute to the eWAT lipolysis by DSS? Our results showed that DSS significantly increased FGF21 level in circulation and tissue expression in the liver and eWAT. Previous studies showed that WAT FGF21 expression might be used locally in adipose tissue for PPAR*γ* activation [31], while liver-produced FGF21 contributes to the elevation of the circulating concentration and exerts its broad functions in various tissues in an endocrine manner [32]. However, incubation of adipocytes with rhFGF21 did not result in an elevated lipolysis suggesting that the role of FGF21 is likely indirect. In fact, DSS-induced circulating cytokines such as IL-6 was earlier than FGF21 induction implying a contribution of IL-6 to FGF21-mediated adipose tissue lipolysis. Indeed, incubating adipocytes with either IL-6 or rhFGF21 did not induce a lipolytic effect, but rhFGF21 significantly induced lipolysis when the adipocytes were pretreated with IL-6. Therefore, the role of FGF21 in the DSS-associated lipolysis is mediated by inflammatory response such as IL-6 elevation. The mechanism(s) behind this regulation warrant further investigation.

## References

[1] Podolsky D. K. (2002). Inflammatory bowel disease. *The New England Journal of Medicine*.

[2] Baumgart D. C., Sandborn W. J. (2012). Crohn’s disease. *Lancet*.

[3] Danese S., Fiocchi C. (2011). Ulcerative colitis. *The New England Journal of Medicine*.

[4] Ling S. C., Griffiths A. M. (2000). Nutrition in inflammatory bowel disease. *Current Opinion in Clinical Nutrition and Metabolic Care*.

[5] Wild G. E., Drozdowski L., Tartaglia C., Clandinin M. T., Thomson A. B. (2007). Nutritional modulation of the inflammatory response in inflammatory bowel disease—from the molecular to the integrative to the clinical. *World Journal of Gastroenterology*.

[6] Rocha R., Santana G. O., Almeida N., Lyra A. C. (2009). Analysis of fat and muscle mass in patients with inflammatory bowel disease during remission and active phase. *The British Journal of Nutrition*.

[7] Capristo E., Mingrone G., Addolorato G., Greco A. V., Gasbarrini G. (1998). Metabolic features of inflammatory bowel disease in a remission phase of the disease activity. *Journal of Internal Medicine*.

[8] Kimmel A. R., Brasaemle D. L., McAndrews-Hill M., Sztalryd C., Londos C. (2010). Adoption of PERILIPIN as a unifying nomenclature for the mammalian PAT-family of intracellular lipid storage droplet proteins. *Journal of Lipid Research*.

[9] Zimmermann R., Strauss J. G., Haemmerle G. (2004). Fat mobilization in adipose tissue is promoted by adipose triglyceride lipase. *Science*.

[10] Villena J. A., Roy S., Sarkadi-Nagy E., Kim K. H., Sul H. S. (2004). Desnutrin, an adipocyte gene encoding a novel patatin domain-containing protein, is induced by fasting and glucocorticoids: ectopic expression of desnutrin increases triglyceride hydrolysis. *The Journal of Biological Chemistry*.

[11] Holm C., Osterlund T. (1999). Hormone-sensitive lipase and neutral cholesteryl ester lipase. *Methods in Molecular Biology*.

[12] Karlsson M., Contreras J. A., Hellman U., Tornqvist H., Holm C. (1997). cDNA cloning, tissue distribution, and identification of the catalytic triad of monoglyceride lipase. Evolutionary relationship to esterases, lysophospholipases, and haloperoxidases. *The Journal of Biological Chemistry*.

[13] Greenberg A. S., Egan J. J., Wek S. A., Moos M. C., Londos C., Kimmel A. R. (1993). Isolation of cDNAs for perilipins A and B: sequence and expression of lipid droplet-associated proteins of adipocytes. *Proceedings of the National Academy of Sciences of the United States of America*.

[14] Egan J. J., Greenberg A. S., Chang M. K., Wek S. A., Moos M. C., Londos C. (1992). Mechanism of hormone-stimulated lipolysis in adipocytes: translocation of hormone-sensitive lipase to the lipid storage droplet. *Proceedings of the National Academy of Sciences of the United States of America*.

[15] Londos C., Brasaemle D. L., Schultz C. J. (1999). On the control of lipolysis in adipocytes. *Annals of the New York Academy of Sciences*.

[16] Inagaki T., Dutchak P., Zhao G. (2007). Endocrine regulation of the fasting response by PPARalpha-mediated induction of fibroblast growth factor 21. *Cell Metabolism*.

[17] Zhao C., Liu Y., Xiao J. (2015). FGF21 mediates alcohol-induced adipose tissue lipolysis by activation of systemic release of catecholamine in mice. *Journal of Lipid Research*.

[18] Potthoff M. J., Inagaki T., Satapati S. (2009). FGF21 induces PGC-1alpha and regulates carbohydrate and fatty acid metabolism during the adaptive starvation response. *Proceedings of the National Academy of Sciences of the United States of America*.

[19] Feingold K. R., Grunfeld C., Heuer J. G. (2012). FGF21 is increased by inflammatory stimuli and protects leptin-deficient ob/ob mice from the toxicity of sepsis. *Endocrinology*.

[20] Gariani K., Drifte G., Dunn-Siegrist I., Pugin J., Jornayvaz F. R. (2013). Increased FGF21 plasma levels in humans with sepsis and SIRS. *Endocrine Connections*.

[21] Kmiec Z. (1998). Cytokines in inflammatory bowel disease. *Archivum Immunologiae et Therapiae Experimentalis (Warsz)*.

[22] Gross V., Andus T., Caesar I., Roth M., Schölmerich J. (1992). Evidence for continuous stimulation of interleukin-6 production in Crohn’s disease. *Gastroenterology*.

[23] Mudter J., Neurath M. F. (2007). Il-6 signaling in inflammatory bowel disease: pathophysiological role and clinical relevance. *Inflammatory Bowel Diseases*.

[24] Peyrin-Biroulet L., Chamaillard M., Gonzalez F. (2007). Mesenteric fat in Crohn’s disease: a pathogenetic hallmark or an innocent bystander?. *Gut*.

[25] Teixeira L. G., Leonel A. J., Aguilar E. C. (2011). The combination of high-fat diet-induced obesity and chronic ulcerative colitis reciprocally exacerbates adipose tissue and colon inflammation. *Lipids in Health and Disease*.

[26] Arner P., Pettersson A., Mitchell P. J., Dunbar J. D., Kharitonenkov A., Rydén M. (2008). FGF21 attenuates lipolysis in human adipocytes - a possible link to improved insulin sensitivity. *FEBS Letters*.

[27] Fiorenza C. G., Chou S. H., Mantzoros C. S. (2011). Lipodystrophy: pathophysiology and advances in treatment. *Nature Reviews. Endocrinology*.

[28] Kolditz C. I., Langin D. (2010). Adipose tissue lipolysis. *Current Opinion in Clinical Nutrition and Metabolic Care*.

[29] Miyoshi H., Souza S. C., Zhang H. H. (2006). Perilipin promotes hormone-sensitive lipase-mediated adipocyte lipolysis via phosphorylation-dependent and -independent mechanisms. *The Journal of Biological Chemistry*.

[30] Haemmerle G., Lass A., Zimmermann R. (2006). Defective lipolysis and altered energy metabolism in mice lacking adipose triglyceride lipase. *Science*.

[31] Dutchak P. A., Katafuchi T., Bookout A. L. (2012). Fibroblast growth factor-21 regulates PPARgamma activity and the antidiabetic actions of thiazolidinediones. *Cell*.

[32] Murata Y., Konishi M., Itoh N. (2011). FGF21 as an endocrine regulator in lipid metabolism: from molecular evolution to physiology and pathophysiology. *Journal of Nutrition and Metabolism*.

